# Emerging non-D_2_ receptor-based therapies for schizophrenia: a focus on muscarinic and glutamatergic pathways

**DOI:** 10.3389/fpsyt.2026.1898212

**Published:** 2026-07-10

**Authors:** Yunxiang Wang, Jiajia Wang, Xiao Zhang, Xudong Liu

**Affiliations:** 1 Department of Clinical Psychology II, Shihezi Oasis Hospital, Shihezi, China; 2 Department of Ultrasound Medicine, The First Affiliated Hospital of Shihezi University, Shihezi, China; 3 Department of Psychiatry IV, Shihezi Oasis Hospital, Shihezi, China; 4 Department of Psychiatry, Shihezi Oasis Hospital, Shihezi, China

**Keywords:** glutamatergic, KarXT, muscarinic receptor, non-D_2_ receptor-based, schizophrenia

## Abstract

Schizophrenia remains a highly disabling neuropsychiatric disorder, particularly due to the paucity of effective therapeutic interventions for negative symptoms and cognitive deficits. For more than seven decades, antipsychotic drug development has been overwhelmingly centered on dopamine D_2_ receptor antagonism. A paradigm shift occurred in 2024 with the FDA approval of KarXT, a first-in-class muscarinic acetylcholine receptor agonist selectively targeting M_1_ and M_4_ subtypes, marking the first non-D_2_ receptor-based antipsychotic approved in over 70 years. This review provides a systematic evaluation of emerging non-D_2_ receptor-based therapeutic strategies for schizophrenia, with particular emphasis on the muscarinic cholinergic pathway (exemplified by KarXT) and the glutamatergic pathway (represented by GlyT1 inhibitors). Phase III clinical trial data demonstrate that KarXT significantly improves both positive and negative symptom domains, exhibits favorable metabolic safety relative to conventional antipsychotics, and shows no evidence of clinically meaningful weight gain or glucose dysregulation. Preliminary evidence from exploratory subgroup analyses suggests potential cognitive benefits in patients with baseline cognitive impairment, but these findings require prospective validation. Long-term safety data remain limited. In contrast, clinical trial outcomes for glutamatergic agents, including GlyT1 inhibitors, have been heterogeneous and inconsistent, leaving the therapeutic viability of this pathway uncertain. Collectively, muscarinic agonism constitutes the first clinically validated non-D_2_ receptor-based mechanism in schizophrenia treatment. Advancing precision psychiatry will require rigorous head-to-head comparative trials and biomarker-informed patient stratification to extend therapeutic benefits beyond psychosis control to functional and cognitive recovery.

## 1 Introduction

Schizophrenia is a severe, chronic, and disabling neuropsychiatric disorder affecting approximately 24 million individuals globally. It is clinically characterized by a triad of symptom domains: positive symptoms (e.g., hallucinations and delusions), negative symptoms (e.g., avolition, anhedonia, and social withdrawal), and cognitive deficits, including impairments in attention, working memory, and executive function (1, 2). While positive symptoms are often the most salient in clinical presentation, cognitive impairment, present in up to 80% of patients, is consistently identified as the strongest predictor of long-term functional disability and real-world outcomes, such as occupational performance, independent living, and social integration (3, 4). Despite its profound impact on functional recovery, cognitive impairment associated with schizophrenia (CIAS) remains a major unmet medical need; to date, no pharmacological agent has received regulatory approval specifically for the treatment of CIAS (5).

For more than seven decades, antipsychotic drug development has been predominantly anchored in dopamine D_2_ receptor antagonism or partial agonism (6, 7). Although first- and second-generation antipsychotics demonstrate robust efficacy against positive symptoms, their effects on negative symptoms and cognitive dysfunction remain modest and clinically insufficient (8, 9). Furthermore, these agents are frequently associated with dose-limiting adverse effects, including extrapyramidal symptoms (EPS), hyperprolactinemia, weight gain, dyslipidemia, and insulin resistance, which collectively compromise treatment adherence, increase risk of relapse, and diminish quality of life (10, 11). Notably, approximately 20–30% of patients meet criteria for treatment-resistant schizophrenia (TRS), defined as inadequate response to at least two adequately dosed and duration-appropriate antipsychotic trials (12). Clozapine, the only antipsychotic approved for TRS, remains underutilized due to its significant safety liabilities, including agranulocytosis, myocarditis, and seizures, necessitating stringent hematological monitoring (13).

These persistent therapeutic gaps have catalyzed intensive investigation into novel, non-D_2_ receptor-based mechanisms of action. A landmark advancement occurred in September 2024, when xanomeline–trospium (KarXT), a first-in-class oral muscarinic M_1_/M_4_ receptor agonist, received FDA approval for the treatment of schizophrenia (14, 15). This approval marked the first regulatory endorsement of a non-D_2_ receptor-based mechanism for schizophrenia in over half a century. Conversely, in January 2025, iclepertin, a highly selective glycine transporter-1 (GlyT1) inhibitor and the most clinically advanced compound targeting the glutamatergic system, failed to meet its primary efficacy endpoints across all three pivotal Phase III trials (the CONNEX program), underscoring the challenges inherent in translating preclinical glutamatergic hypotheses into clinical success (16, 17).

This review synthesizes recent advances in non-D_2_ receptor-based therapeutic strategies for schizophrenia, with particular emphasis on muscarinic and glutamatergic modulation. We provide a critical appraisal of the current clinical evidence, address unresolved mechanistic and methodological controversies, and propose a forward-looking framework for the development of precision-targeted interventions that extend beyond dopamine D_2_ receptor blockade.

## 2 Limitations of dopamine-based treatments

All currently approved antipsychotics exert their effects primarily by antagonizing or partially activating the dopamine D2 receptor (6, 7). Although they are effective in treating positive symptoms, their limitations have become increasingly evident.

First-generation antipsychotics effectively alleviate positive symptoms but have little effect on negative symptoms and may even exacerbate them through excessive dopamine blockade in the mesocortical pathway (8, 18). Second-generation antipsychotics moderately improve negative symptoms and have a lower risk of EPS, but their efficacy for cognitive impairment remains negligible (19). A recent systematic review and network meta-analysis confirmed that antipsychotics are not cognitive enhancers; the modest improvement in cognitive performance observed compared with placebo likely reflects secondary benefits from the improvement of positive symptoms rather than genuine cognitive enhancement (5).

Approximately 20–30% of patients meet criteria for treatment-resistant schizophrenia (12). Clozapine is the only drug approved for this population, but due to its side effect profile, including agranulocytosis (requiring routine blood monitoring) and metabolic disturbances, it remains underutilized (13). Moreover, even among clozapine-treated patients, approximately 30–40% show insufficient response (20).

Notably, clozapine’s pharmacology extends well beyond dopamine D_2_ receptor antagonism. Clozapine and its major metabolite, N-desmethylclozapine (norclozapine), exhibit complex interactions with the cholinergic system, including actions at M1 and M4 muscarinic receptors. This has led to the hypothesis that clozapine’s unique efficacy in treatment-resistant schizophrenia may be partially attributable to muscarinic receptor modulation, an idea that has gained renewed interest following the approval of KarXT. However, this hypothesis remains controversial: a recent critical review argues that clozapine’s intrinsic muscarinic activity is relatively weak, norclozapine has limited brain penetration, and the effect sizes observed with KarXT are modest relative to clozapine’s clinical effects, collectively questioning muscarinic mechanisms as the primary explanation for clozapine’s superiority. Regardless of the ultimate mechanism, the conceptual convergence between clozapine and KarXT at the muscarinic receptor level underscores the therapeutic potential of this pathway and highlights the need for further investigation into whether selective muscarinic agonists could offer benefits in treatment-resistant populations (48).

Traditional antipsychotics are associated with severe side effects. First-generation agents frequently cause EPS, including potentially irreversible tardive dyskinesia (10). Second-generation agents are associated with significant metabolic disturbances, including weight gain and dyslipidemia (11). These side effects contribute to poor medication adherence, with non-adherence rates of approximately 40–50% reported in meta-analyses (21).

In summary, the limitations of dopamine-based treatments—limited efficacy for negative and cognitive symptoms, high rates of treatment resistance, and substantial side effect burdens—have driven the search for non-D_2_ receptor-based mechanisms.

## 3 Emerging non-D_2_ receptor-based mechanisms

### 3.1 Muscarinic pathway: M1/M4 receptor agonism (KarXT)

The cholinergic system has long been implicated in the pathophysiology of schizophrenia, particularly in the domains of cognitive dysfunction and negative symptoms. Among the five muscarinic acetylcholine receptor subtypes (M1–M5), M1 and M4 receptors have emerged as the most therapeutically promising targets for next-generation antipsychotics. The M1 receptor is predominantly expressed in the prefrontal cortex and hippocampus, where it modulates synaptic plasticity, learning, and memory. In contrast, the M4 receptor is highly enriched in striatal regions and functions as a heteroreceptor that negatively regulates dopamine release, thereby influencing psychotic symptom expression (22, 23). Preclinical evidence consistently demonstrates that selective activation of M1 and M4 receptors elicits antipsychotic-like effects and enhances cognitive performance in rodent models of schizophrenia (24–26).

The most clinically advanced agent targeting this pathway is KarXT, a fixed-dose oral combination of xanomeline (a centrally active, functionally selective M1/M4 agonist) and trospium chloride (a peripherally restricted muscarinic antagonist). Xanomeline was initially evaluated as monotherapy but development was halted due to dose-limiting peripheral cholinergic adverse effects, primarily gastrointestinal disturbances (27). Trospium chloride does not cross the blood–brain barrier and selectively antagonizes peripheral muscarinic receptors, thereby mitigating these side effects without compromising central target engagement or pharmacodynamic efficacy (28).

In September 2024, KarXT received FDA approval for the treatment of schizophrenia in adults, representing the first antipsychotic with a primary mechanism of action not involving direct dopamine D_2_ receptor blockade approved in over seven decades (14, 15). This regulatory decision was grounded in robust clinical evidence from the EMERGENT phase III program. In the pivotal EMERGENT-2 trial, KarXT demonstrated statistically significant and clinically meaningful reductions in total Positive and Negative Syndrome Scale (PANSS) scores relative to placebo at week 5 (least squares mean difference: −9.6; 95% CI: −13.9 to −5.2; *P* < 0.0001; Cohen’s d = 0.61) (29). Comparable efficacy was observed in EMERGENT-3, with a PANSS reduction of −8.4 (95% CI:−12.4 to −4.3; *P* < 0.001; d = 0.60) (30). Improvements were evident across both positive and negative symptom subscales, with effect sizes ranging from 0.48 to 0.66 (31).

A recent individual-participant-data meta-analysis integrating results from three randomized controlled trials (N = 674) confirmed the consistency and magnitude of KarXT’s efficacy: the pooled mean difference in PANSS total score versus placebo was −9.71 (95% CI: −12.33 to −7.09; I² = 0%), with parallel improvements in positive (MD = −3.21) and negative (MD = −1.62) symptom subscales (32). Significant improvement was also observed in Clinical Global Impression–Severity (CGI-S) scores (MD = −0.58), reinforcing the clinical relevance of these findings.

Beyond symptomatic control, exploratory evidence suggests possible cognitive benefits observed in an exploratory subgroup of patients treated with KarXT, but these findings remain preliminary. In a prespecified *post-hoc* integrated analysis of EMERGENT-2 and EMERGENT-3, KarXT significantly improved cognitive performance among patients with baseline cognitive impairment defined as ≥1 standard deviation below normative population means (Cohen’s d = 0.54; p = 0.004) (33). Using a more stringent threshold (≤ −1.5 SD), the effect size increased further (d = 0.80). Notably, correlations between cognitive improvement and concurrent changes in PANSS scores were negligible, suggesting that KarXT’s cognitive-enhancing effects may operate independently of its antipsychotic activity. However, it is critical to emphasize that these cognitive findings derive exclusively from retrospective, exploratory subgroup analyses. As the authors themselves note, these observations require prospective validation using standardized, domain-specific cognitive assessments in stable outpatients with prespecified cognitive impairment criteria. At present, KarXT is not indicated for cognitive impairment associated with schizophrenia (CIAS), and no conclusion can be drawn regarding its cognitive effects in patients without baseline impairment.

Regarding safety, KarXT demonstrates a favorable tolerability profile. The most frequently reported treatment-emergent adverse events are mild-to-moderate, transient gastrointestinal disturbances, including nausea (19–22%), constipation (13–21%), dyspepsia (16–19%), and vomiting (14–16%) (29, 30, 34). Importantly, the incidence of extrapyramidal symptoms (EPS), weight gain, and sedation was comparable to placebo, distinguishing KarXT mechanistically and clinically from conventional dopaminergic antipsychotics (29, 34). A systematic review and meta-analysis encompassing all three pivotal RCTs corroborated KarXT’s advantageous metabolic and motor safety profile, reporting no statistically significant differences versus placebo in EPS incidence or body weight change (34).

Despite these encouraging outcomes, several limitations warrant consideration. First, the cognitive benefits observed to date derive exclusively from retrospective subgroup analyses of acutely ill, hospitalized patients; prospective trials enrolling stable outpatients with prospectively confirmed, clinically meaningful cognitive impairment are needed. Second, all registrational trials were of short duration (5 weeks), limiting conclusions regarding long-term efficacy maintenance and safety. Third, the ARISE trial, a Phase 3 study assessing KarXT as adjunctive therapy to established atypical antipsychotics (NCT04738123), failed to meet its primary endpoint. According to the sponsor’s announcement, KarXT added to ongoing antipsychotic treatment did not demonstrate a statistically significant reduction in PANSS total score compared with placebo plus antipsychotic at week 5 (primary endpoint). The least squares mean difference was reported as −2.9 (95% CI not disclosed; p = 0.19), with effect sizes substantially smaller than those observed in monotherapy trials (35). Several explanations for this negative result warrant consideration. First, the substantial efficacy of background atypical antipsychotics may have left limited additional improvement to be captured by KarXT, creating a ceiling effect. Second, potential pharmacodynamic interactions between muscarinic M1/M4 agonism and dopamine D2 antagonism cannot be ruled out, as these pathways may converge on overlapping downstream signaling cascades. Third, the short 5-week treatment duration may have been insufficient to observe the full effects of adjunctive therapy, particularly if muscarinic and dopaminergic mechanisms require different temporal windows to achieve synergistic effects. Regardless of the underlying explanation, this finding indicates that the incremental benefit of adding KarXT to ongoing dopaminergic treatment remains uncertain, and KarXT is currently indicated only as monotherapy.

While KarXT represents a landmark achievement, it is important to recognize that xanomeline activates multiple muscarinic receptor subtypes (M1–M5), making it a relatively non-selective tool. This has prompted efforts to develop next-generation compounds with improved subtype selectivity. Selective M4 positive allosteric modulators (PAMs) have shown promising preclinical results: repeated dosing with VU0467154 in mice enhances cognitive performance and produces antipsychotic-like effects without evidence of tolerance (26). These observations, together with the cognitive benefits observed in exploratory subgroup analyses of KarXT, suggest that optimal therapeutic outcomes may require dual M1/M4 activation rather than selective M4 agonism alone. Next-generation compounds capable of engaging both M1 and M4 receptors with improved selectivity are currently in preclinical and early clinical development, though clinical data are not yet available (**Table 1**).

**Table 1:** Summary of novel pharmacological approaches for schizophrenia.

Compound	Mechanism	Stage	Key efficacy findings	Key safety/tolerability	Important limitations
KarXT	M1/M4 agonist	FDA approved (2024)	PANSS Δ = -9.6 to -8.4; d=0.60-0.61	GI events; no EPS/weight gain	Cognitive benefit only in exploratory *post-hoc* subgroup analysis (impaired patients; d=0.54-0.80); requires prospective validation
Iclepertin	GlyT1 inhibitor	Phase III failed	MCCB: no difference vs placebo (p=0.63)	Favorable	Complete lack of cognitive efficacy despite promising Phase II data; trial assay sensitivity compromised by practice effects and placebo response

Data from (17, 29, 30, 32, 33).

### 3.2 Glutamatergic pathway: GlyT1 inhibitors and NMDA regulation

The glutamate hypothesis of schizophrenia posits that NMDA receptor hypofunction underlies the pathophysiology of the disorder, providing a compelling rationale for developing glutamatergic agents (36, 37). Glycine transporter 1 (GlyT1) inhibition enhances NMDA receptor function by increasing synaptic glycine concentration, making it a promising approach (38).

Iclepertin (BI 425809) is the most advanced GlyT1 inhibitor specifically developed for cognitive impairment associated with schizophrenia (CIAS). Phase II studies demonstrated encouraging results: in a 12-week trial (NCT02832037, n = 509), iclepertin at 10 mg and 25 mg once daily produced statistically significant improvements in the MATRICS Consensus Cognitive Battery (MCCB) overall composite T-score compared with placebo (39). However, in January 2025, iclepertin failed to meet its primary and key secondary endpoints in the Phase III CONNEX program (16).

The CONNEX program comprised three identically designed, multinational, randomized, double-blind, placebo-controlled trials conducted at 338 specialist psychiatric centers across 41 countries. A total of 1,835 patients received at least one dose of trial medication (918 iclepertin, 917 placebo), with 1,602 completing the 26-week treatment period (17). The primary endpoint, change from baseline to week 26 in MCCB overall composite T-score, showed no statistically significant difference between iclepertin and placebo in any individual trial or in the pooled population (adjusted mean difference 0.127, 95% CI: −0.396 to 0.650; p = 0.63) (17).

A critical observation from the CONNEX program was the magnitude of placebo response. While placebo responses at week 12 were comparable between Phase II and Phase III, the placebo group showed additional improvement at week 26 in Phase III, consistent with practice effects. The defining feature distinguishing Phase II from Phase III outcomes was the substantially smaller cognitive improvement with iclepertin in Phase III, suggesting that repeated testing had a greater effect on MCCB performance than iclepertin itself (17).

The failure of iclepertin is not an isolated event. Earlier GlyT1 inhibitors, including bitopertin, also failed to replicate Phase II findings (40). Luvadaxistat, a DAAO inhibitor, failed to replicate positive Phase II cognitive signals and was discontinued in September 2024 (41, 42).

Several mechanistic explanations have been proposed for this failure. Singer and Yee (43) suggested that astrocytic GlyT1 blockade may disrupt the serine shuttle mechanism, deplete activity-dependent glycine release, or perturb the cyclic operation of GlyT1. These insights underscore the complexity of glycine neurotransmission and the importance of neuron–glia interactions (43).From a translational perspective, these mechanistic considerations imply that simply increasing ambient glycine levels may be insufficient to restore NMDA receptor function in a temporally or spatially appropriate manner, potentially explaining why pharmacological GlyT1 inhibition has failed to replicate preclinical promise in clinical settings.

The failure of iclepertin should not be interpreted as definitive refutation of all NMDA receptor approaches. Pomaglumetad, a metabotropic glutamate receptor 2/3 agonist being redeveloped using precision medicine methods, illustrates that glutamatergic strategies may require biomarker-guided patient selection (44).

**Figure 1:**
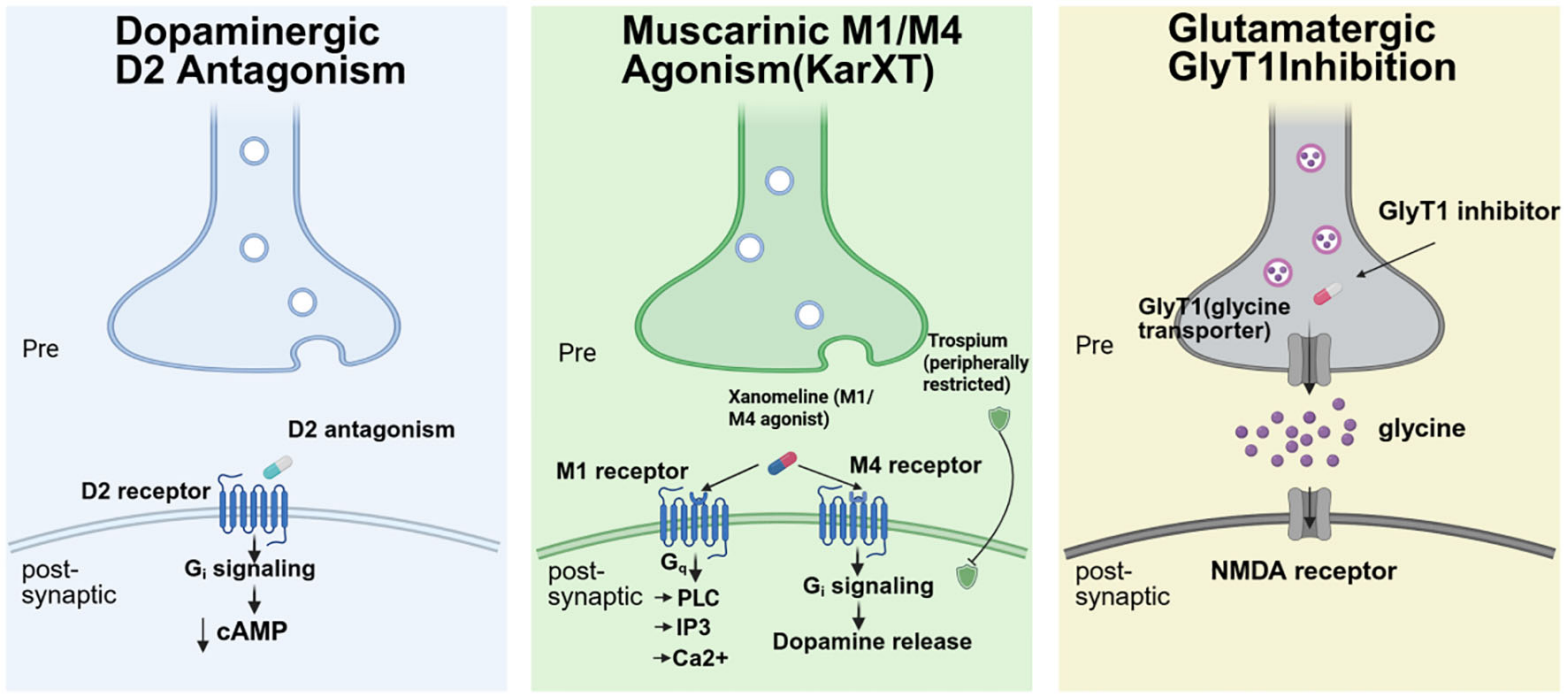
Schematic comparison of dopaminergic and emerging non-D_2_ receptor-based mechanisms in schizophrenia. Left panel: Traditional antipsychotics target D2 receptors, activating Gi signaling and reducing cAMP, providing efficacy for positive symptoms (green check) but having minimal effects on negative symptoms and no effect on cognitive deficits. Middle panel: KarXT (xanomeline/trospium) activates M1/M4 receptors, triggering Gq/Gi cascades and improving both positive and negative symptoms (double green check); the antipsychotic effect is mediated indirectly through M4 receptor activation, which reduces mesolimbic dopaminergic activity;trospium reduces gastrointestinal side effects; cognitive benefits remain exploratory and require prospective validation (dashed box). Right panel: GlyT1 inhibitors (iclepertin) aim to enhance NMDA function but the Phase III CONNEX program failed (p=0.63); all clinical benefits unconfirmed (red question mark). Dashed lines indicate unconfirmed or failed efficacy.

A schematic comparison of the three mechanistic pathways discussed above—dopaminergic D2 antagonism, muscarinic M1/M4 agonism, and glutamatergic GlyT1 inhibition—is presented in **Figure 1**, which highlights their distinct downstream signaling cascades and divergent clinical profiles. While the muscarinic pathway has successfully translated into an approved therapy, the glutamatergic pathway remains clinically unvalidated despite robust preclinical rationale, underscoring the translational challenges inherent in CNS drug development.

## 4 Challenges and future directions

The landscape of schizophrenia drug development has been fundamentally reshaped by the FDA approval of KarXT and the failure of iclepertin (16). Although KarXT validated non-D_2_ receptor-based mechanisms, the failure of iclepertin revealed a systematic pattern of translational failure. This section explores the key challenges and outlines priority research areas for the future.

### 4.1 The systematic challenge of escalating placebo responses

A key FDA analysis documented the fundamental transformation of schizophrenia trial dynamics over the past 15 years (45). Before 2009, the average placebo response was −6.4 PANSS points, and the treatment effect was −8.6 points. After 2009, the placebo response increased to −10.5 PANSS points (a 64% increase), while the treatment effect dropped to −5.8 points (a 33% decrease). Trial success rates fell from 78% to 57% (45). North American trials showed the most significant increase, with placebo responses rising from −4.3 to −8.5 points and treatment effects dropping by 62% (45).

### 4.2 Why phase II success leads to phase III failure: the scaling problem

A meta-regression analysis by Kantrowitz et al. (46) examined 159 comparisons across 13,000 participants, demonstrating that placebo responses scale disproportionately with increasing study size and number of sites, while active treatment responses remain stable. This differential scaling results in systematic effect-size deflation in larger multicenter trials. Statistical significance is most reliably achieved in smaller studies with no more than 150 participants, fewer sites, and higher recruitment density per site (46).

### 4.3 Biomarker development for patient stratification

The failure of multiple promising compounds in late-stage trials highlights the critical need for validated biomarkers for patient stratification (47). The precision medicine approach of pomaglumetad, which uses genomic markers to identify treatment responders, exemplifies the potential of biomarker-guided redevelopment (44). For glutamatergic approaches, biomarkers that distinguish patients with primarily glycine-dependent versus D-serine-dependent NMDA function could inform patient selection (41, 43).

Trace Amine-Associated Receptor 1 (TAAR1). TAAR1 is a G protein-coupled receptor widely expressed in monoaminergic brain regions, including the ventral tegmental area, substantia nigra, and prefrontal cortex. It is activated by endogenous trace amines and modulates dopaminergic, serotonergic, and glutamatergic neurotransmission (49). Preclinical evidence has demonstrated that TAAR1 activation produces antipsychotic-like effects without inducing catalepsy (49). However, recent living systematic reviews indicate that the TAAR1 agonists ulotaront and ralmitaront showed few differences compared with placebo in acute schizophrenia trials, with large placebo responses contributing to the failure (50). Despite these setbacks, TAAR1 remains a mechanistically distinct target that bypasses D_2_ receptor blockade, and ongoing research continues to explore next-generation TAAR1 agonists (49).

### 4.4 Excellence in trial design and operation

Priority areas for methodological reform include: focusing on fewer, higher-quality sites with greater recruitment density; implementing centralized rating systems to reduce inter-site variability; using power calculations that account for effect-size deflation; and adopting adaptive trial designs that allow early identification and exclusion of sites with aberrant placebo responses (46).For example, sponsors should prioritize site selection based on historical recruitment performance and rater reliability metrics rather than geographic distribution. Centralized remote rating using video-recorded interviews has been shown to reduce inter-site variability by 30-40% in recent trials. Adaptive designs that allow early termination of sites with excessive placebo responses could preserve assay sensitivity without increasing sample size (**Table 2**).

**Table 2:** Lessons learned from failed schizophrenia drug development programs.

Compound	Mechanism	Phase of failure	Key lesson learned	Reference(s)
Iclepertin	GlyT1 inhibitor	Phase III	Practice effects from repeated cognitive testing can obscure drug effects; patient enrichment based on baseline impairment may be necessary	(16, 17)
Luvadaxistat	DAAO inhibitor	Phase II	Inverted U-shaped dose-response requires careful dose selection; high cognitive measure variability complicates signal detection	(41, 42)
Bitopertin	GlyT1 inhibitor	Phase III	Phase II efficacy signals may not replicate in larger trials; negative symptoms are difficult to target selectively	(40)
Emracidine	M4 PAM	Phase II	High placebo responses (13.5–16.1 PANSS points) eliminated drug–placebo separation; site selection and rater training are critical	(1)

Synthesized lessons:

1. Patient enrichment (baseline cognitive impairment, biomarker-defined subgroups) is essential.

2. Trial design (fewer high-quality sites, centralized rating, adaptive designs) must evolve.

3. Outcome measures need to account for practice effects and real-world functioning.

4. Placebo response is the single biggest challenge; controlling it requires methodological rigor, not larger sample sizes.

## 5 Conclusion

Schizophrenia drug development has reached an inflection point where methodological innovation matters as much as mechanistic innovation. The FDA approval of KarXT—which achieves antipsychotic efficacy through indirect, M_4_-mediated downregulation of mesolimbic dopamine activity—validates non-D_2_ receptor-based mechanisms and demonstrates that when mechanistic plausibility is aligned with optimized trial methodology, success is more likely. KarXT significantly improves both positive and negative symptoms and has a favorable metabolic and motor profile. Exploratory *post-hoc* analyses suggest potential procognitive effects in a subgroup of patients with baseline cognitive impairment (Cohen’s d=0.54–0.80), but these findings require prospective confirmation in dedicated trials using standardized, domain-specific cognitive assessments. At present, KarXT has not demonstrated conclusive cognitive benefits in the general schizophrenia population and is not approved for cognitive impairment associated with schizophrenia.

**Figure 2:**
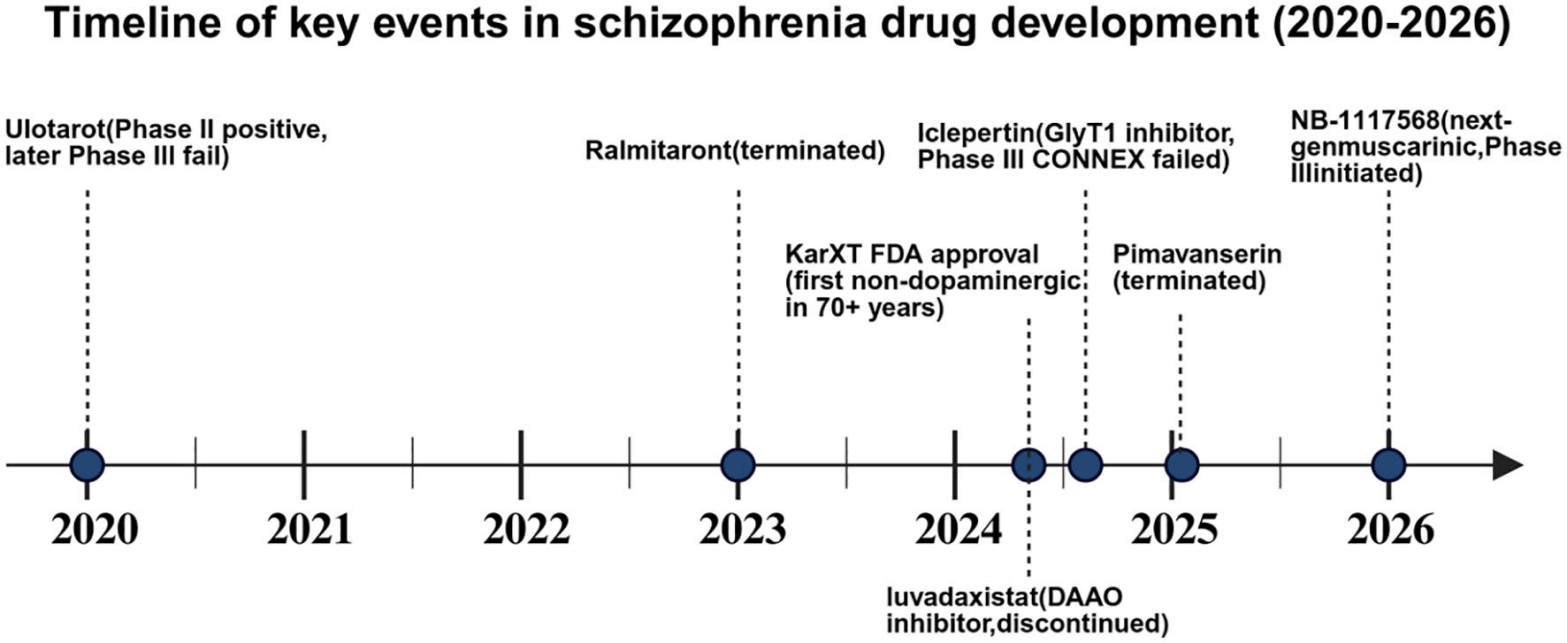
Timeline of key events in schizophrenia drug development (2020–2026). KarXT FDA approval (September 2024) — first antipsychotic with a non-D_2_ receptor-based mechanism approved in over 70 years. Failures (gray circles): ulotaront positive Phase II (2020) followed by Phase III failure; ralmitaront termination (2023); luvadaxistat discontinuation (September 2024); iclepertin Phase III CONNEX failure (January 2025, p=0.63); pimavanserin termination (March 2025). Ongoing (blue circle): NBI-1117568 Phase III initiation (2026). The concurrent success and repeated failures underscore the need for methodological reform. Abbreviations: DAAO, GlyT1, TAAR1, 5-HT2A.

The broader landscape of schizophrenia drug development from 2020 to 2026 is summarized in **Figure 2**, which illustrates the concurrent validation of non-D_2_ receptor-based mechanisms through KarXT (FDA approval, September 2024) and the systematic failure of multiple mechanistically sound compounds, including iclepertin (January 2025), luvadaxistat (September 2024), and pimavanserin (March 2025). This timeline reinforces a central thesis of this review: mechanistic innovation alone is insufficient; methodological reform in trial design is equally critical for successful translation.

Conversely, the failure of iclepertin reveals a systematic pattern of translational failure. The erosion of trial assay sensitivity due to escalating placebo responses has become a rate-limiting factor. As demonstrated by the Kantrowitz meta-regression, placebo responses scale disproportionately with increasing trial size and number of sites, resulting in systematic effect-size deflation.

Three strategic priorities emerge. Precision medicine frameworks must replace broad population approaches: biomarkers are essential for identifying subgroups most likely to benefit from specific mechanisms. Outcome measures must evolve beyond symptom rating scales to capture real-world functioning. Trial methodology urgently requires reform: fewer high-quality sites, higher recruitment density, centralized rating systems, and power calculations that account for differential placebo scaling.

Muscarinic agonism constitutes a validated non-D_2_ receptor-based mechanism for the treatment of schizophrenia, achieving its effects through indirect modulation of dopaminergic signaling. Future head-to-head trials and biomarker-driven patient stratification are essential for achieving precision pharmacotherapy that extends beyond psychotic symptoms. However, without substantial methodological reforms, mechanistically sound compounds will continue to fail in late-stage trials.Despite these challenges, the approval of KarXT demonstrates that non-D_2_ receptor-based mechanisms can succeed when mechanistic rationale is paired with rigorous trial methodology, offering new hope for patients who have not responded to traditional antipsychotics.
